# Genomic regions responsible for seminal and crown root lengths identified by 2D & 3D root system image analysis

**DOI:** 10.1186/s12864-018-4639-4

**Published:** 2018-04-20

**Authors:** Yusaku Uga, Ithipong Assaranurak, Yuka Kitomi, Brandon G. Larson, Eric J. Craft, Jon E. Shaff, Susan R. McCouch, Leon V. Kochian

**Affiliations:** 1 Institute of Crop Science, National Agriculture and Food Research Organization, 2-1-2 Kannondai, Tsukuba, Ibaraki 305-8518 Japan; 2 Department of Plant Breeding and Genetics, Cornell University, Ithaca, NY 14853 USA; 3 Department of Global Agricultural Sciences, Graduate School of Agricultural and Life Sciences, The University of Tokyo, Yayoi, Bunkyo, Tokyo, 113-8657 Japan; 4 Robert W. Holley Center for Agriculture and Health, USDA-ARS, Cornell University, Ithaca, NY 14853 USA; 5 Global Institute for Food Security, University of Saskatchewan, Saskatoon, Canada

**Keywords:** Chromosome segment substitution line (CSSL), Image processing, *Oryza sativa*, QTL, Root phenotyping, Root system architecture

## Abstract

**Background:**

Genetic improvement of root system architecture is a promising approach for improved uptake of water and mineral nutrients distributed unevenly in the soil. To identify genomic regions associated with the length of different root types in rice, we quantified root system architecture in a set of 26 chromosome segment substitution lines derived from a cross between lowland *indica* rice, IR64, and upland *tropical japonica* rice, Kinandang Patong, (IK-CSSLs), using 2D & 3D root phenotyping platforms.

**Results:**

Lengths of seminal and crown roots in the IK-CSSLs grown under hydroponic conditions were measured by 2D image analysis (RootReader2D). Twelve CSSLs showed significantly longer seminal root length than the recurrent parent IR64. Of these, 8 CSSLs also exhibited longer total length of the three longest crown roots compared to IR64. Three-dimensional image analysis (RootReader3D) for these CSSLs grown in gellan gum revealed that only one CSSL, SL1003, showed significantly longer total root length than IR64. To characterize the root morphology of SL1003 under soil conditions, SL1003 was grown in Turface, a soil-like growth media, and roots were quantified using RootReader3D. SL1003 had larger total root length and increased total crown root length than did IR64, although its seminal root length was similar to that of IR64. The larger TRL in SL1003 may be due to increased crown root length.

**Conclusions:**

SL1003 carries an introgression from Kinandang Patong on the long arm of chromosome 1 in the genetic background of IR64. We conclude that this region harbors a QTL controlling crown root elongation.

**Electronic supplementary material:**

The online version of this article (10.1186/s12864-018-4639-4) contains supplementary material, which is available to authorized users.

## Background

The acquisition of water and nutrients from the soil by plant root systems is essential for the survival and vigor of terrestrial plants. Appropriate root development and distribution is important for the efficient acquisition of both water and nutrients, because these resources are often distributed unevenly in the soil [1, 2]. Adequate distribution of plant root systems is even more important for plant growth under marginal soil conditions. In general, a deeper root system is beneficial under drought conditions, in order to acquire water at depth, as water is the most mobile nutrient [3]. Additionally, a shallower root system has been shown to be advantageous for acquisition of relatively immobile nutrients such as phosphorus, as the phosphate anion tends to be fixed on clay minerals in the topsoil [4]. The distribution of root systems in the vertical dimension for monocotyledonous crop plants such as maize and rice is mainly determined by the root growth angle (RGA) and maximum root length (MRL) of seminal and crown roots [5, 6]. Shallow versus steep RGAs promote preferential root distribution in the topsoil and subsoil, respectively. Additionally, small and large MRLs are associated with compact and more extensively distributed root system architectures (RSAs), respectively.

Improvement of productivity in rice, a staple food for nearly half of the world’s population, is required to meet the demand of an increasing world population. However, global climate change combined with economic development in recent years has exacerbated the pressure on scarce water and mineral nutrient resources for rice production. Under such conditions, improvements in efficient acquisition of these resources by optimizing RSA for specific microenvironments will be essential for steady increases in crop production in the future [7]. Previous studies have reported quantitative trait loci (QTLs) for root traits associated with RSA such as RGA and MRL in several crops. In rice, our group detected six QTLs for RGA on chromosomes 2, 4, 6, 7, and 9 in different mapping populations [8–12]. Lou et al. [13] found six rice QTLs associated with RGA located on chromosomes 1, 2, 4, 7, and 10. Two rice QTLs for root gravitropic response, a component of RGA, were identified on chromosomes 6 and 10 [14]. Among all of these rice RSA QTL, *DRO1*, which has been detected on chromosome 9 in recombinant inbred lines (RILs) derived from a cross between the lowland cultivar ‘IR64’ and the upland cultivar ‘Kinandang Patong’, has been the first to be cloned and the resulting information used for molecular breeding of improved drought avoidance [15]. On the other hand, more than 100 QTLs for root length have been reported distributed across all of the rice chromosomes [16–32]. To date, no QTLs responsible for root length have been cloned in rice, although some QTLs for MRL have recently been fine-mapped in rice. For example, *qRL1.1* was mapped on chromosome 1 using a population of Taichung 65 (*Oryza sativa*) / IRGC 104038 (*O. glaberrima*) back cross recombinant lines, *qRL6.1* was mapped on chromosome 6 using Koshihikari / Kasalath chromosome segment substitution lines (CSSLs) at the seedling stage under hydroponic conditions [30, 31], and *qRL7* was mapped on chromosome 7 using Xiequingzao / R9308 RILs at heading stage under hydroponic conditions [33].

Monocotyledonous crop plants have a root system that consists of one or more seminal roots that originate from the seed embryo after germination and crown roots that later emerge from nodes along the stem [34]. Seminal roots have a key role in absorption of water and nutrients at the early growth stage, whereas crown roots become more important for acquisition of these resources at later growth stages [2]. Therefore, it is important to consider both root types concurrently in the context of plant growth when trying to improve uptake efficiency of water and nutrients through molecular breeding for root length. However, for QTL analyses of the length of the longest roots in a root system, MRL, the research has been conducted without discriminating between seminal and crown root growth. Therefore, it is unclear whether elongation of seminal and crown roots is controlled by the same or different genetic mechanisms. Additionally, total root length (TRL) of the root system as well as MRL may be important traits for the uptake efficiency of water and nutrients. However, there have been few reports on TRL QTL in rice [35, 36].

In this study, to detect chromosomal regions associated with the elongation of seminal and/or crown roots in rice, we measured seminal root length (SRL) and crown root length (CRL) in a population of CSSLs grown in hydroponic culture using two-dimensional (2D) analysis of root system images. From these measurements, several lines with longer SRL and CRL were identified. Typical hydroponic culture of plants does not allow for maintenance of three-dimensional (3D) RSA. Therefore, we grew these lines in transparent gellan gum cylinders, using the methods of Clark et al. [37], in order to determine if SRL and/or CRL QTL from the 2D analysis of hydroponically grown plants co-located in the rice genome with other root architecture related traits. Finally, to determine if QTLs for root length and RSA traits identified under gel condition exist when rice plants are grown in soil or soil-like growth media, we also grew the rice plants using a specially constructed plastic mesh to allow root growth to maintain 3D RSA in Turface, a soil-like growth media, using the methods described in Piñeros et al. [38].

## Methods

### Plant materials

To identify genomic regions associated with natural variation for root length in cultivated rice, we used a set of 26 CSSLs derived from a cross between IR64 and Kinandang Patong (IK-CSSLs in the IR64 genetic background; Uga et al. [11]). IR64 is a modern lowland cultivar (*indica*; IRGC 66970) developed by the International Rice Research Institute in the Philippines, and is widely grown in South and Southeast Asia. Kinandang Patong (KP) is a traditional upland cultivar (*tropical japonica*; IRGC 23364) that originated in the Philippines.

### Measurement of root traits in the 26 IK-CSSLs grown under hydroponic conditions by 2D-image-based phenotyping

Seeds were soaked in 70% ethanol for 1 min and then washed with sterilized water 3 times. Those seeds were sterilized in 4.5% sodium hypochlorite for 30 min and then washed with sterilized water 5 times. The sterilized seeds were germinated in moistened germination paper rolls (Anchor Paper, St. Paul, MN, USA) for 5 days at 30 °C in an incubator with illuminated light conditions. Uniformly grown seedlings were selected and transferred into a hydroponic growth system consisting of black plastic tubs (L × W × H = 500 × 360 × 220 mm) filled with modified Magnavaca’s nutrient solution, pH 5.5 [39]. Seedlings were positioned in black, closed-cell polyethylene foam strips (McMaster-Carr, Elmhurst, IL, USA) which served to support seedling growth during the experiment and prevent illumination of the nutrient solution, as described previously [35]. Twelve seedlings were grown in each strip (L × W × H = 360 × 50 × 8 mm) with 2.5-cm spacing between seedlings. Seedlings were grown in a walk-in growth chamber for the duration of the experiment (Environmental Growth Chambers, Inc., Chagrin Falls, OH; 28 °C day / 24 °C night, 14 h / 10 h photoperiod, 790-800 μmol of light intensity). The nutrient solution was replaced with fresh nutrient solution every 2 days, at which time we re-randomized both tubs in the growth chamber and strips in the tubs. The pH was maintained at 5.5 by adjustment with KOH or HCl every day.

The root system of each seedling was spread with water in a specimen tray and photographed using a digital camera (Nikon D200 with a 60 mm Micro lens, Nikon Inc., Melville, NY, USA) interfaced to a personal computer. To measure seminal and crown root lengths on each root system, digital root images were analyzed using the RootReader2D software [35]. We measured only the first, second and third longest crown roots (1st CRL to 3rd CRL) because many crown roots that developed during the late seedling stage overlapped with each other in the 2D images, making the short, fine crown roots difficult to measure. To determine shoot and root dry weights, samples were harvested at 15 days after germination (DAG), dried in an oven at 80 °C for 3 days and weighed.

To compare the root traits of IR64 and Kinandang Patong, we measured 20 plants from each accession (10 plants in each strip × 2 strips), excluding the outside two rows in each strip to avoid edge effects. To evaluate root traits of the 26 IK-CSSLs, we measured ten plants per line (10 plants in each foam strip), excluding the outside two rows in each strip. Because up to seven strips could be set in each hydroponic tub, four tubs were employed for this experiment.

### Measurement of root traits in 8 selected IK-CSSLs grown in gellan gum media by 3D-image-based phenotyping

To quantify three-dimensional root system architectural development in 8 IK-CSSLs, we used a gellan-gum growth system as described by Clark et al. [37]. Seeds were dehulled and soaked in 70% ethanol for 1 min, washed with sterilized water 3 times, sterilized in 4.5% sodium hypochlorite for 30 min, and then washed with sterilized water 5 times. The sterile seeds were sown and germinated in square petri-dishes with modified Magnavaca’s media (0.15% gellan gum, Sigma-Aldrich, St Louis, MO, USA). The petri dishes were placed for 30 to 32 h at 30 °C under dark conditions in an incubator. Once the seeds germinated and the radicle had emerged and grown to approximately 1 cm in length, uniform seedlings were transferred into glass growth cylinders (dia. × H = 90 × 280 mm; MicroGlass, Kiowa, CO, USA) containing 1.3 L of modified Magnavaca’s medium (pH 5.5) solidified by gellan gum. The gellan gum medium was prepared by dissolving and autoclaving 1.95 g of gellan gum powder in 0.65 L of distilled water. The sterile gellan gum solution was then combined with 0.65 L of 2 × strength modified Magnavaca’s solution that was adjusted to pH 5.5 and passed through a sterilized filter, as described previously [37]. After transplanting seedlings into the cylinders, the top of each cylinder was covered by a black plastic cap with a small air hole and the side of the cylinder was wrapped with opaque plastic to prevent light penetration. Seedlings were grown in a growth chamber for the duration of the experiment (30 °C day / 26 °C night, 12 h /12 h photoperiod, 1000 μmol of light intensity). The experiments were organized in a completely randomized design with 2 replicates. A total of 9 samples (first replicate = 5 plants, second replicate = 4 plants) was used to calculate line means.

The root system of each seedling was photographed in the gellan gum cylinders using a digital camera (Nikon D300s with a 180 mm lens, Nikon Inc., Melville, NY, USA) interfaced to a personal computer, as described previously [37]. Forty 2D digital images per plant were taken as the cylinder containing the root system was rotated through 360° (a 2D image taken every 9° of rotation). The 40 2D images were reconstructed into a 3D root system image using RootReader3D software [37] and a suite of root architecture traits were automatically calculated. In this study, we analyzed three RSA traits using RootReader3D: total root length (cm), centroid (cm; centroid is distance from center of mass of the root system to the seed), and maximum root depth (cm). The root growth angle of each seedling at 11 DAG was determined by measuring the angle between the medium surface (horizontal line) and the shallowest crown root, as described previously [15], using US National Institutes of Health ImageJ software. At 15 DAG, plants were removed from the gellan gum, root and shoot samples were washed and measured for root number and shoot length, and then dried in an oven at 80 °C for 3 days and weighed to determine shoot and root dry weights.

### Measurement of root traits in SL1003 grown in Turface under flooded conditions using 3D-image-based phenotyping

For evaluation of three-dimensional root morphology in SL1003 under soil-like conditions, plants were grown in coarse Turface clay (Profile Products LLC., Buffalo Grove, IL, USA), which is a soil-like growth medium, instead of natural soil. Seeds were soaked in 70% ethanol for 1 min, washed with sterilized water 3 times, sterilized in 4.5% sodium hypochlorite for 30 min and then washed with sterilized water 5 times. The sterilized seeds were germinated in moistened germination paper rolls for 3 days at 30 °C in an incubator with illuminated light conditions. Uniform seedlings were transferred into Turface growth media, as described by Piñeros et al. [38]. The Turface growth system included an ABS plastic mesh tower (dia. × H = 130 × 250 mm) made within a 3D printer MakerBot Replicator 2X (MakerBot Industries, Brooklyn, NY, USA) that was placed into a PVC cylinder (dia. × H = 150 × 300 mm). Six cylinders were placed into plastic tubs (L × W × H = 610 × 300 × 300 mm). The tubes containing the plastic mesh towers were filled with Turface up to the top mesh layer of the mesh tower (Additional file 1: Figure S1A). Modified Magnavaca’s nutrient solution (pH 5.5) was then poured into the cylinder up to the surface of the Turface. Seedlings were grown in Turface under aerated hydroponic conditions in a growth chamber for the duration of the experiment (30 °C day / 26 °C night, 12 h / 12 h photoperiod, 1000 μmol of light intensity; Additional file 1: Figure S1B). The nutrient solution was changed every 2 days. The experiments were organized in a completely randomized design with 2 replicates. A total of 10 samples (first replicate = 6 plants, second replicate = 4 plants) was used to calculate line means.

Imaging and phenotyping of roots grown in the Turface system was carried out as described for sorghum plants grown in the same plastic mesh tower system in hydroponic media as described in Hufnagel et al. [40], except in this case the Turface was removed from the mesh tower holding the plant root system before root system imaging (Additional file 1: Figure S1C). For each plant, the root system grown through the mesh tower was carefully placed in the water-filled imaging tank (Additional file 1: Figure S1D). Then, 100 2D digital images were taken as the plant and root system was rotated through 360° (a 2D image was taken every 3.6° of rotation). After removing the image of the colored mesh from original digital images, the 100 2D images were reconstructed into a 3D root system image using RootReader3D. In this study, we analyzed three RSA traits for each plant at 15 DAG using RootReader3D: total root length (cm), centroid (cm), and maximum root depth (cm). After imaging the root systems, seedlings were carefully taken out from the mesh tower and other root traits were measured: primary root length of seminal and all crown roots (cm), total length of primary roots (calculated by adding primary root lengths of seminal and all crown roots, cm), total root number, shoot length (cm), and dry weights of shoot and roots.

## Results

### Root traits in the 26 IK-CSSLs grown under hydroponic conditions

**Fig. 1:**
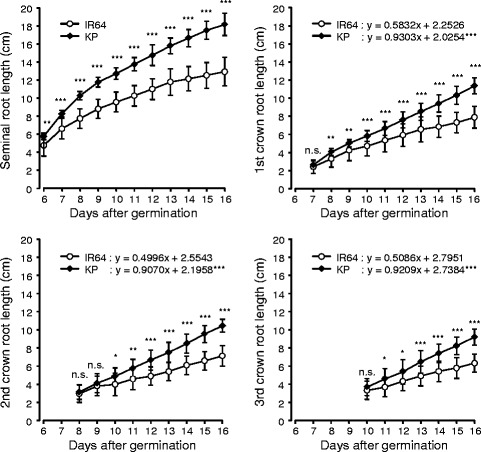
Time course for root development between IR64 and Kinandang Patong (KP) grown in hydroponic media. Plot shows mean ± s.d. (*n* = 20). ^*^Significant at 5% level, ^**^Significant at 1% level, ^***^Significant at 0.1% level, n.s. Not significant (Student’s *t*-test)

A time course for root growth showed that the SRL of KP was significantly longer than that of IR64, starting at 6 DAG (Fig. 1). The 1st CRL, 2nd CRL, and 3rd CRL in KP were significantly longer than in IR64 at 8, 10, and 11 DAG, respectively. The 1st to 3rd crown roots in each cultivar showed similar root growth rates although the time of emergence of each crown root was different, suggesting that growth rates of crown roots are similar regardless of developmental stage in the rice plant (Fig. 1).

**Fig. 2:**
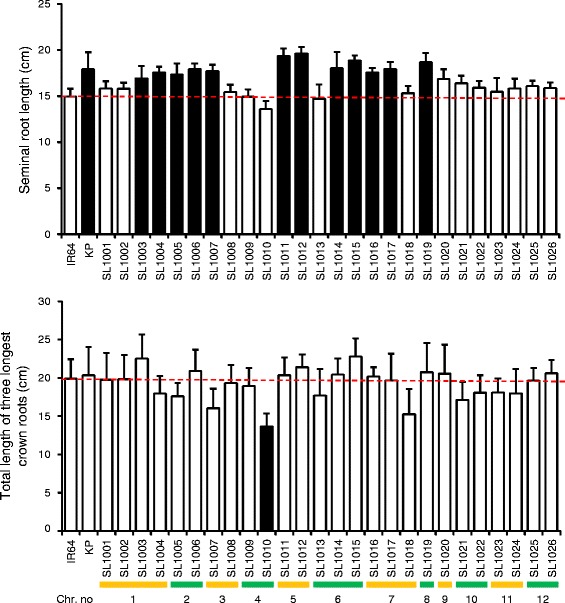
Lengths of seminal and crown roots for the 26 IK-CSSLs grown in hydroponic media. Root traits were collected at 14 days after germination. Values are means + s.d. (*n* = 10). The black histograms in the plots of seminal root length and total length of three longest crown roots differ significantly between the IK-CSSL’s and IR64 (*p* < 0.001, Dunnett’s test). Dashed red line indicates mean root length values for IR64. Bottommost lines and numbers indicate chromosomal location of Kinandang Patong (KP) introgression in the IR64 background

Twelve of the 26 CSSLs showed significantly longer SRLs (16.85 to 19.62 cm) at 14 DAG than IR64 (14.97 cm) (Fig. 2). SL1011 and SL1012 had longer SRL than IR64 at all developmental stages measured (Additional file 2: Figure S2). Only SL1010 had significantly smaller total length of the three longest crown roots (TL3LCR) compared to IR64 (Fig. 2). On the other hand, ten CSSLs exhibited greater TL3LCR compared to IR64, although these values were not significant (Fig. 2). The TL3LCR was moderately correlated with SRL (*r* = 0.575) (Table 1). We also found that TL3LCR was more highly correlated with shoot length (*r* = 0.652) and shoot dry weight (*r* = 0.714) compared to SRL, suggesting that crown root development is more important for shoot development after the late seedling stage than seminal root development. Among the 26 CSSLs, only SL1003, SL1004, and SL1006 were taller (shoot length) than IR64 (Additional file 3: Figure S3). No CSSLs showed significantly larger shoot and root dry weight than IR64 plants (Additional file 3: Figure S3). However, all root traits showed a positive correlation with shoot traits (Table 1).

**Table 1:** Coefficients of correlation among root and shoot traits in the IK-CSSLs

	SRL		TL3LCR		RDW		SL	
TL3LCR	0.575	***						
RDW	0.529	***	0.647	***				
SL	0.428	*	0.652	***	0.523	***		
SDW	0.557	***	0.714	***	0.882	***	0.5604	***

*SRL* seminal root length, *TL3LCR* total length of three longest crown roots, *RDW* root dry weight, *SL* shoot length, *SDW* shoot dry weight

*p < 0.05; ***p < 0.001

### Root traits in 8 selected IK-CSSLs grown in gellan gum media

**Fig. 3:**
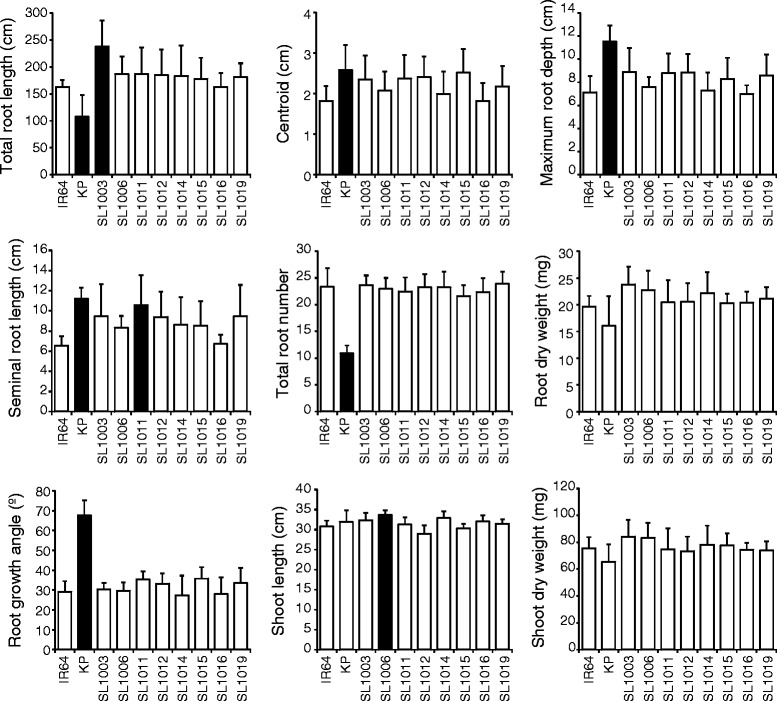
Root and shoot traits measured for 8 IK-CSSLs grown in gellan gum media. Total root length (total lengths of primary and lateral roots in seminal and crown roots), centroid, and maximum root depth data were collected at 11 days after germination; other traits, 15 days after germination. Values are means + s.d. (*n* = 9). Black bars differ significantly between the IK-CSSL and IR64 (*p* < 0.05, Dunnett’s test)

To validate the root phenotypes of the eight selected CSSLs that had longer SRL and CRL than IR64, we measured root traits of seedlings grown in gellan gum media using 3D RSA image analysis. Only SL1003 had significantly longer TRL than IR64 (46% longer; Fig. 3). It is noteworthy that KP had shorter TRL than IR64 because KP had a smaller total root number. SRLs of all lines grown in gellan gum were inhibited and showed wider variation of their SRLs compared to those of the same rice genotypes grown in hydroponic solution (Figs. 2 and 3). Only SL1011 had significantly longer SRL in gellan gum than IR64. For the other five root traits measured (centroid, maximum root depth, total root number, root dry weight, and root growth angle), all CSSLs showed similar phenotypes to IR64 (Fig. 3).

**Fig. 4:**
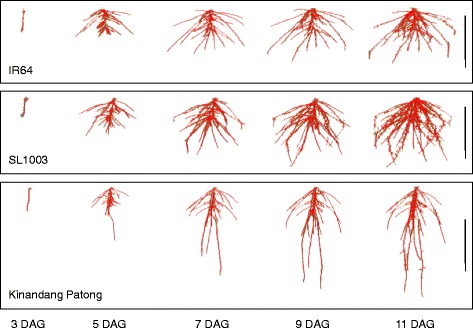
3D root system models in SL1003, IR64, and Kinandang Patong grown in gellan gum media. Each representative 3D model was reconstructed using the RootReader3D software. DAG: days after germination. Scale bars, 5 cm

Evaluation of TRL in all CSSLs revealed that SL1003 TRL was significantly greater than IR64 starting at 7 DAG (Additional file 4: Figure S4). However, the total root number of SL1003 was similar to that of IR64 (Fig. 3) and the SRL of SL1003 was only slightly larger than IR64. Therefore, the significant increase in TRL in SL1003 appears to be due to increased crown root and lateral root length, which is suggested from the 3D reconstructions of RSA for IR64, SL1003 and KP depicted in Fig. 4.

### Root length in SL1003 grown in Turface under flooded conditions

**Fig. 5:**
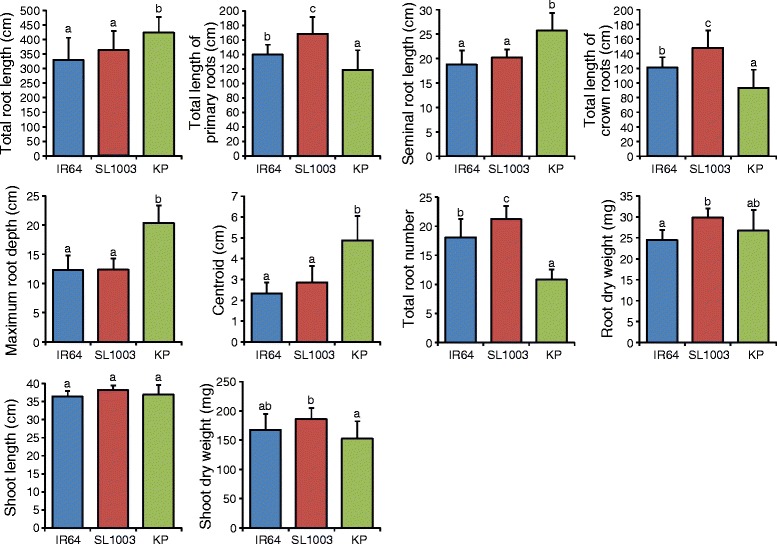
Root and shoot traits for SL1003, IR64, and Kinandang Patong (KP) grown in Turface. All traits were collected at 15 days after germination. Values are means + s.d. (IR64 and KP, *n* = 10; SL1003, *n* = 8). Values labeled with different letters differ significantly among the 3 lines (*p* < 0.05, Tukey’s HSD test)

To clarify whether the root phenotypes of SL1003 observed in the gellan gum system are also observed in a soil environment, we grew the same lines in a soil-like media consisting of Turface clay particles (Additional file 1: Figure S1; Additional file 5: Video S1; Additional file 6: Video S2 and Additional file 7: Video S3). The mean TRL of SL1003 was 32 cm longer than that of IR64. This value was not significant because standard deviation for the mean TRL of each line in Turface was larger than in gellan gum (Fig. 5). However, the size of the entire root system for SL 1003 appears to be larger than that of IR64. So, we investigated possible differences in TRL between SL1003 and IR64. When only primary roots without lateral roots were quantified, total length of the primary root in SL1003 was significantly greater (29 cm greater total length of primary roots) than in IR64 (Fig. 5). Also, SL1003 exhibited significantly greater length of the crown roots (total crown root length was 27 cm longer) than in IR64 and had a significantly greater number of roots than IR64. However, the SRL of SL1003 was not significantly different than IR64. The other measured root traits, maximum root depth and centroid, were not significantly different between SL1003 and IR64 (Fig. 5).

**Fig. 6:**
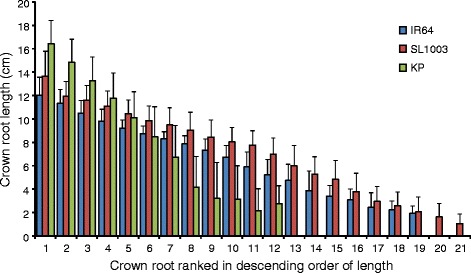
Length of crown roots for SL1003, IR64, and Kinandang Patong (KP) grown in Turface. All traits were collected at 15 days after germination. Values are means + s.d. (*n* = 3 to 10). Numbers along x-axis indicate specific crown roots ranked in descending order of length. e.g. #1 represents mean of longest crown root in each line

To elucidate whether the difference in TRL in SL1003 was due primarily to increased length of crown roots or increased total root number, we measured the length of each crown root individually in all plants. All crown roots in SL1003 were longer than those in IR64 (Fig. 6). There were two additional crown roots in SL1003 that did not have counterparts in IR64, indicated as the 20th and 21th crown roots in Fig. 6. However, these were the smallest roots, measuring only a few centimeters, and the increased number of crown roots did not explain the increased total primary root length of crown roots in SL1003. Thus, we conclude that the increased TRL of SL1003 is due to longer root length of each crown root compared to IR64.

## Discussion

### 3D phenotyping platform as a powerful tool to dissect RSA

In this study, there were several factors limiting what we could accomplish including throughput limitations which set a limit on number of root systems we could image per day. Therefore, we selected the 8 CSSLs for more in depth study with an appropriate number of biological replicates based on the results of the higher throughput 2D root system image analysis before investigating RSA of CSSLs via 3D image analysis. The 2D image analysis using root samples grown in hydroponic conditions allowed us to take images of many root samples each day [35]. However, the three-dimensionally-developed root system had to be spread in a specimen tray and many roots overlapped with each other on the 2D image. This was especially true for the numerous crown roots in a root system, where the significant root overlap in the older rice seedlings probably resulted in an underestimation of total root length. Therefore, we measured only seminal roots and the three longest crown roots which were clearly distinguishable on the 2D images and could be readily analyzed using RootReader2D. On the other hand, RootReader3D can automatically quantify TRL from the 3D reconstruction of the root system, although it was also difficult to measure the length of each separate crown root in the older seedlings because of the same problem of some roots obscuring view of other roots. 3D RSA analysis also allows us to quantify root traits that measure more complex root architecture traits such as centroid and maximum root depth, which could be obtained only from root systems where the 3D architecture is maintained.

### Detection of chromosome regions associated with SRL and CRL

To identify chromosomal regions associated with natural variation of root length, we measured root traits in the 26 CSSLs derived from the cross between IR64 and Kinandang Patong using 2D and 3D root phenotyping platforms. Twelve CSSLs had significantly longer SRLs than IR64 under hydroponic growth conditions (Fig. 2), suggesting that QTLs for SRL are located on chromosomes 1, 2, 3, 5, 6, 7, and 8. Eight of those twelve CSSLs also had longer CRLs, while the other four (SL1004, SL1005, SL1007, and SL1017) were not different than IR64. These results indicate that four regions on chromosomes 1, 2, 3, and 7 harbor QTLs specifically associated with elongation of seminal roots rather than crown roots. A study by Courtois et al. [41] reported QTLs for root length on all chromosomes. We compared the relationship between chromosomal segments associated with root length in this study and QTL positions reported in previous studies based on the Nipponbare genome sequence in the Rice Annotation Project database [42]. Only one chromosomal region, from 10.52 to 11.07 Mbp on chromosome 6 in SL1014, was not previously identified as carrying QTL for root length, although *qRL6.2* (RM6119 to RM7023, 4.43 to 6.97 Mbp) was located near this region [31].

Of the eight CSSLs grown under gellan gum conditions, SL1003 showed the largest TRL compared to IR64 (Fig. 3; Additional file 4: Figure S4). When SL1003 was grown in soil-like conditions using Turface, SL1003 also exhibited increased TRL compared to IR64. This was due to increased primary root length of crown roots, rather than increases in other root traits such as lateral root length of crown roots, seminal root length, or total root number (Figs. 5 and 6). Additionally, SL1003 was not significantly different than IR64 for other shoot and root traits such as shoot length, maximum root depth, and centroid. These findings suggest that SL1003 has robust QTL(s) associated with elongation of crown roots which could function under soil-like as well as gel and hydroponic growth conditions. We designate this QTL as *qCRL1* (*quantitative trait locus for Crown Root Length 1*). On the other hand, the shoot length of SL1003 was longer than that of IR64 grown in hydroponic growth conditions, suggesting that this phenomenon might also be influenced by differences in culture conditions between hydroponic and solid media.

### Effect of qCRL1 on root development in different growth conditions

TRL in SL1003 was increased by 46% and 10% compared to IR64 TRL when grown in gel and soil-like media, respectively, indicating that the absolute TRL of SL1003 differed substantially in the two media. Although increased TRL in SL1003 in Turface was associated with increased length of individual crown roots, this was not seen when the same lines were grown in gellan gum. Instead, it appears that growth in gellan gum was associated with increased lateral root length growth on both seminal and crown roots as well as increased crown root growth in SL1003 grown compared to IR64, based on reconstructions of 3D RSA (Fig. 4). However, it was not possible to directly quantify which root types contributed to the increased TRL in SL1003 due to limitations in the version of RootReader3D used for this study.

It is likely that the gellan gum root growth media is less aerated than the Turface growth system. Clark et al. [37] noted that the growth of the rice root system was less in gellan gum compared to growth in hydroponic or sand culture systems, possibly due to somewhat less oxygen availability. Piñeros et al. [38] observed that IR64 plants grown in Turface showed less elaborated, less branched, and more tortuous root systems than those in gellan gum, probably due to thigmotropic responses when the root tip encounters relatively impenetrable clay particles. The difference in TRL in SL1003 when grown in gel vs. soil-like media might be due to root plasticity for oxygen availability and/or the physical resistance of the growth medium. To determine whether this phenomenon resulted from a pleiotropic effect of the *qCRL1* or other QTLs carried on the chromosome segment derived from Kinandang Patong in SL1003, we are currently developing genetic materials to fine-map *qCRL1*.

### Relationship between qCRL1 and other root QTLs

**Fig. 7:**
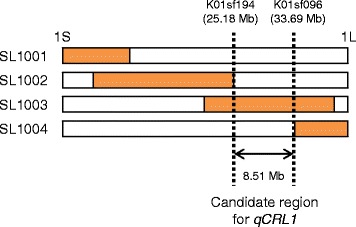
The candidate region for *qCRL1* on chromosome 1. White and orange boxes represent regions that are homozygous for IR64 and Kinandang Patong, respectively. S, short arm; L, long arm. K01sf194 and K01sf096 are SNP marker names and the numbers in parentheses under them indicate their physical map positions (Mb) in the ‘Nipponbare’ genome based on the latest version of the RAP database (http://rapdb.dna.affrc.go.jp)

According to the graphical genotypes of the 26 IK-CSSLs [11], *qCRL1* is located in the region between SNP marker K01sf194 (25.18 Mbp) and K01sf096 (33.69 Mbp) on chromosome 1 (Fig. 7). The size of this candidate region is around 8.5 Mbp in the ‘Nipponbare’ genome, based on the latest version of the RAP database [42]. Although there is no report of QTL specifically for crown root length in this interval, we scanned the literature to identify other QTLs related to root length previously reported in the region. We found many reported QTLs for root length [18, 19, 23, 25, 28] in this interval. Obara et al. [31] mapped *qRL1.1*, a QTL for root length of seedlings grown hydroponically with sufficient NH_4_^+^, located between SSR markers RM6648 (32.34 Mbp) and RM5407 (36.38 Mbp). They mentioned that an aspartate aminotransferase gene, *OsAAT2* (Os01g0760600), is a candidate for *qRL1.1* because of its proximity and function, suggesting that it might be related to regulation of seminal root elongation. Many QTLs for other root traits such as root number, root thickness, and root dry weight were also located in this region, suggesting that this chromosomal region includes robust QTL(s) for root development. Since we did not measure the complete suite of traits associated with root architecture in IR64 and SL1003, it is possible that *qCRL1* has pleiotropic effects on other root architecture traits. Further study using a near-isogenic line (NIL) for *qCRL1* should be conducted to clarify whether *qCRL1* has pleiotropic effects on other root morphology and/or architecture traits.

## Conclusions

The 2D and 3D root phenotyping platforms used in this study allowed us to identify several genomic regions associated with root system development in rice. Among them, the region on the long arm of chromosome 1 that includes *qCRL1* was identified. *qCRL1* appears to be a robust QTL for crown root length detected under hydroponic, gel, and soil-like growth conditions. We speculate that this QTL detected in multi-environmental conditions could be a useful resource for genetic improvement of root system architecture. To do so, it is necessary to validate the effect of *qCRL1* under natural field conditions using its NIL. In this study, we also found other genomic regions associated with root development in IK-CSSLs grown under hydroponic conditions. Since we have not yet evaluated root system architecture of these lines grown in Turface, some lines showing potential root phenotypes might be found from the phenotyping additional CSSLs in the Turface growth system by using 3D root image analysis. Further analysis using the Turface growth system will be beneficial for the identification of new genetic resources for root system architecture.

## Additional files

Additional file 1: **Figure S1.** Sequential stages of constructing the Turface root growth and phenotyping system. (PDF 142 kb)

Additional file 2: **Figure S2.** Time course for seminal root growth and the growth of the 1st to 3rd longest crown roots for the 26 IK-CSSLs, IR64, and Kinandang Patong, grown in hydroponic media. (PDF 2853 kb)

Additional file 3: **Figure S3.** Root and shoot dry weights, and shoot length at 15 days old (15 DAG) plants for the 26 IK-CSSLs, IR64, and Kinandang Patong grown in hydroponic media. (PDF 332 kb)

Additional file 4: **Figure S4.** Time course for of total root growth for the 8 IK-CSSLs, IR64, and Kinandang Patong grown in gellan gum media. (PDF 854 kb)

Additional file 5: **Video S1.** Representative root systems at 15 days after germination for IR64 grown in Turface supplied with nutrient solution. (AVI 1345 kb)

Additional file 6: **Video S2.** Representative root systems at 15 days after germination for SL1003 grown in Turface supplied with nutrient solution. (AVI 1439 kb)

Additional file 7: **Video S3.** Representative root systems at 15 days after germination for Kinandang Patong grown in Turface supplied with nutrient solution. (AVI 1780 kb)

## Abbreviations

2D: Two dimensional
3D: Three dimensional
CRL: Crown root length
CSSLs: Chromosome segment substitution lines
DAG: Days after germination
MRL: Maximum root length
QTL: Quantitative trait locus
RGA: Root growth angle
RSA: Root system architecture
SRL: Seminal root length
TL3LCR: Total length of the three longest crown roots
TRL: Total root length
