# Supplementary material for: Genomic regions responsible for seminal and crown root lengths identified by 2D & 3D root system image analysis
Source: BMC Genomics. 2018 Apr 20;19:273. doi: 10.1186/s12864-018-4639-4 (PMC5910583; doi:10.1186/s12864-018-4639-4)
Supplement: Supplementary file 1 — Figure S1. Sequential stages of constructing the Turface root growth and phenotyping system. (PDF 142 kb) [file 12864_2018_4639_MOESM1_ESM.pdf]

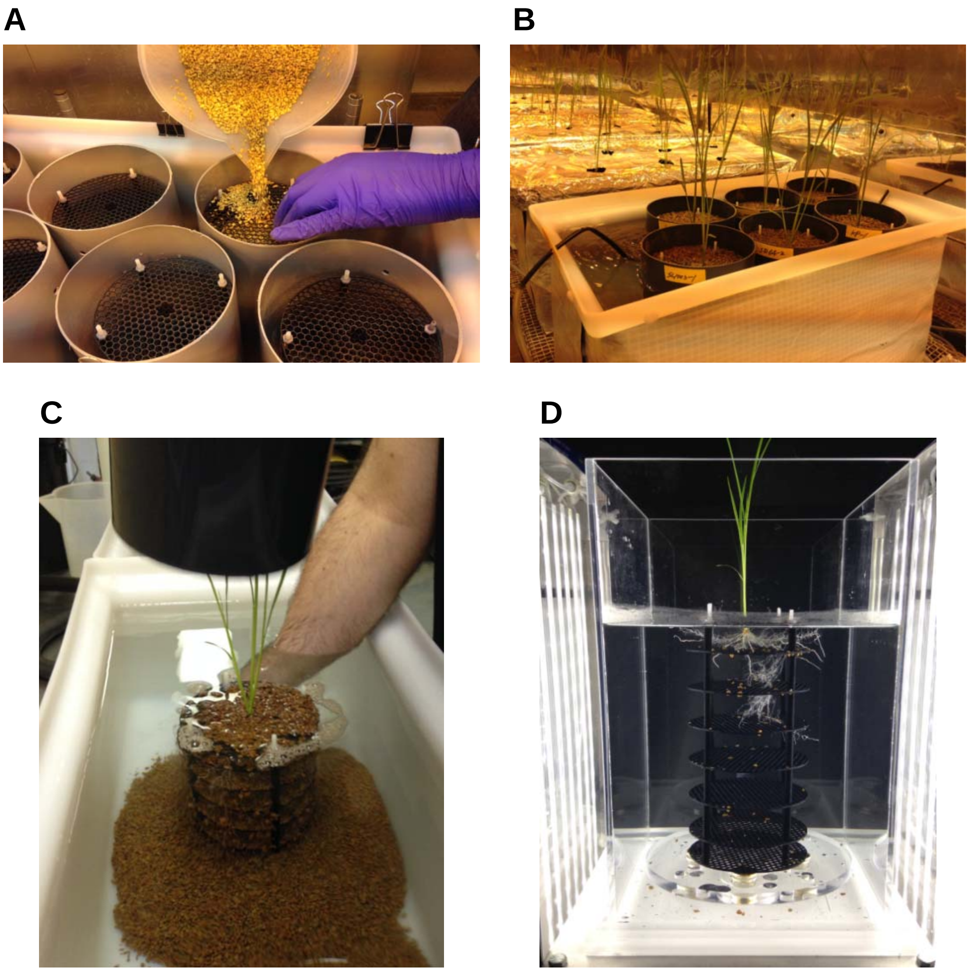

Figure S1 Sequential stages of constructing the Turface root growth and phenotyping system. (A) Filling Turface in PVC tube with mesh tower. (B) Growing rice plants under Turface with flooded conditions. (C) Removing Turface from mesh tower in a tub. (D) Imaging root system of each plant in a water tank.
