# Supplementary material for: Genomic regions responsible for seminal and crown root lengths identified by 2D & 3D root system image analysis
Source: BMC Genomics. 2018 Apr 20;19:273. doi: 10.1186/s12864-018-4639-4 (PMC5910583; doi:10.1186/s12864-018-4639-4)
Supplement: Supplementary file 2 — Figure S2. Time course for seminal root growth and the growth of the 1st to 3rd longest crown roots for the 26 IK-CSSLs, IR64, and Kinandang Patong, grown in hydroponic media. (PDF 2853 kb) [file 12864_2018_4639_MOESM2_ESM.pdf]

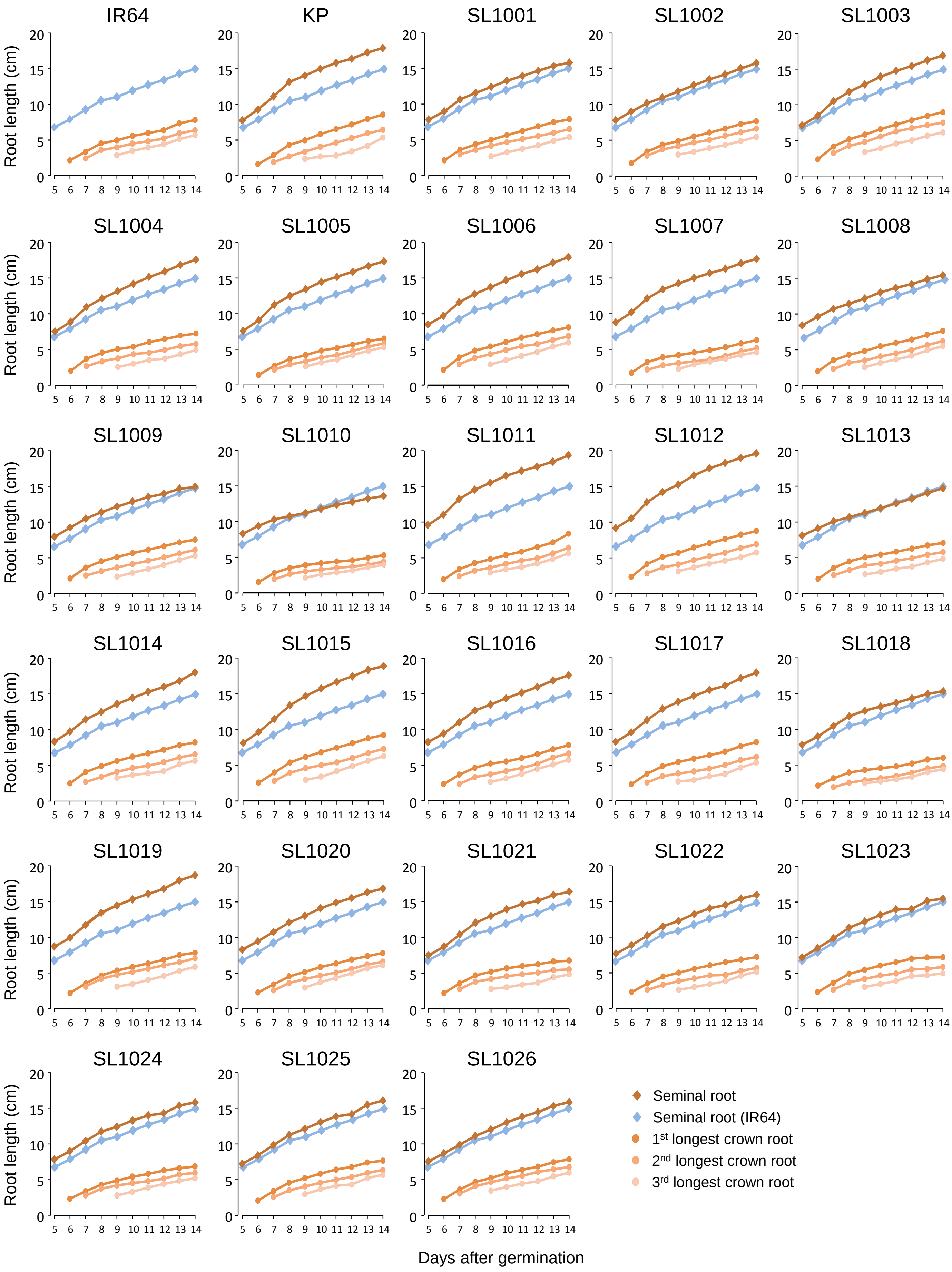

Figure S2 Time course for seminal root growth and the growth of the 1st to 3rd longest crown roots for the 26 IK-CSSLs, IR64, and Kinandang Patong (KP), grown in hydroponic media. Plot shows mean (n = 10).
