# Supplementary material for: Genomic regions responsible for seminal and crown root lengths identified by 2D & 3D root system image analysis
Source: BMC Genomics. 2018 Apr 20;19:273. doi: 10.1186/s12864-018-4639-4 (PMC5910583; doi:10.1186/s12864-018-4639-4)
Supplement: Supplementary file 3 — Figure S3. Root and shoot dry weights, and shoot length at 15 days old (15 DAG) plants for the 26 IK-CSSLs, IR64, and Kinandang Patong grown in hydroponic media. (PDF 332 kb) [file 12864_2018_4639_MOESM3_ESM.pdf]

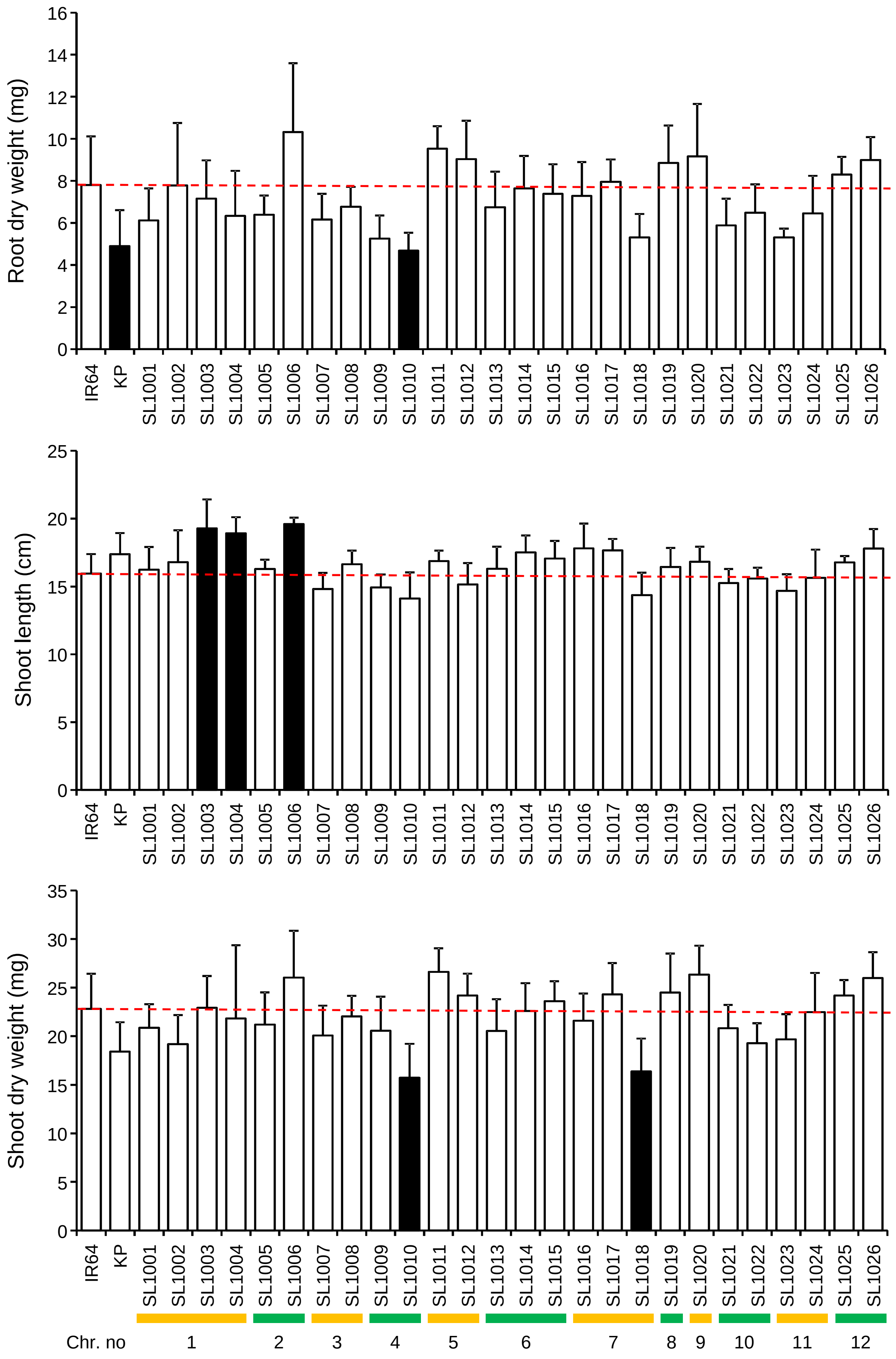

Figure S3 Root and shoot dry weights, and shoot length at 15 day old plants for the 26 IK-CSSLs, IR64, and Kinandang Patong (KP) grown in hydroponic media. Values are means + s.d. (n = 10). Black bars differ significantly between the IK-CSSL and IR64 ($p <$ 0.01, Dunnett's test). Dashed red lines present mean values in IR64.
