# Supplementary material for: Genomic regions responsible for seminal and crown root lengths identified by 2D & 3D root system image analysis
Source: BMC Genomics. 2018 Apr 20;19:273. doi: 10.1186/s12864-018-4639-4 (PMC5910583; doi:10.1186/s12864-018-4639-4)
Supplement: Supplementary file 4 — Figure S4. Time course for of total root growth for the 8 IK-CSSLs, IR64, and Kinandang Patong grown in gellan gum media. (PDF 854 kb) [file 12864_2018_4639_MOESM4_ESM.pdf]

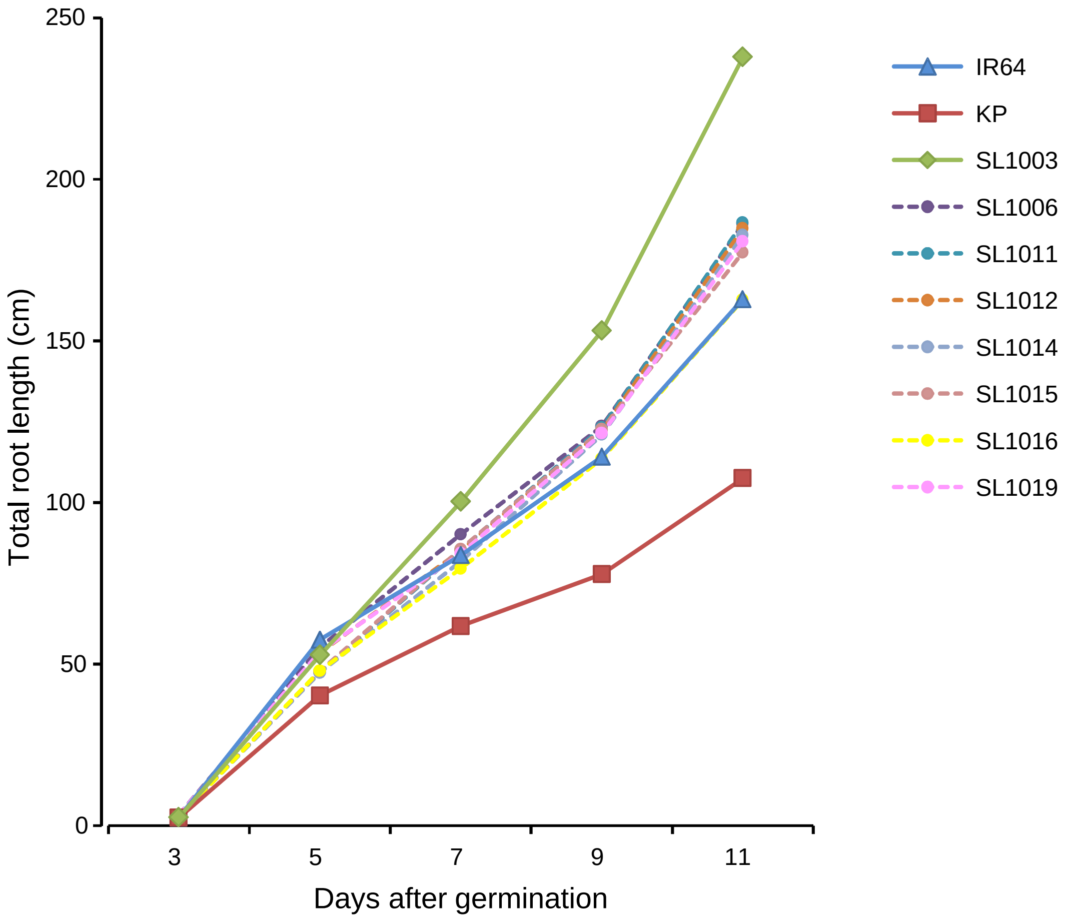

Figure S4 Time course for of total root growth for the eight IK-CSSLs, IR64, and Kinandang Patong (KP) grown in gellan gum media. Plot shows mean (n = 9).
